# Virtual Reality as a Pain Control Adjunct in Orthopedics: A Narrative Review

**DOI:** 10.7759/cureus.66401

**Published:** 2024-08-07

**Authors:** Trisha Vuong, Kai Zhu, Andrew Pastor

**Affiliations:** 1 Orthopedics, Washington State University Elson S. Floyd College of Medicine, Everett, USA; 2 Orthopedic Surgery, Washington State University Elson S. Floyd College of Medicine, Spokane, USA; 3 Orthopedics, Everett Clinic, Edmonds, USA

**Keywords:** surgery, orthopedics, post operative, pain control, virtual reality

## Abstract

Orthopedic surgeons typically prescribe opioids for postoperative pain management as they are effective in managing pain. However, opioid use can lead to issues such as overdose, prescription excess, inadequate pain management, and addiction. Virtual reality (VR) therapy is an alternative route for postoperative pain management that has grown in popularity over the years. VR therapy involves immersing patients in a virtual 3D experience that is anticipated to alleviate pain. In this review article, we summarized the findings of numerous PubMed studies on the effectiveness of VR therapy for postoperative pain control. VR therapy is beneficial for reducing anxiety, pain, and opioid use after surgical procedures across various specialties. Further studies should explore VR therapy in orthopedic procedures.

## Introduction and background

Postoperative pain control is a critical component of postoperative patient recovery. Prescription opioids are effective for treating both acute and chronic pain [1,2]. However, the Centers for Disease Control and Prevention (CDC) has reported a growing epidemic of opioid-related deaths [3]. Between 1999 and 2021, 280,000 people died from prescription opioids [3]. In 2021 alone, 17,000 deaths related to prescription opioid overdoses were reported [3]. The high prevalence of opioid-related deaths makes pain management challenging for postoperative patients.

In orthopedic surgery, procedures involve the manipulation of musculoskeletal tissues, bones, and tendons [4]. The nature of these procedures makes orthopedic surgery one of the most painful procedures compared to other specialties [5]. Orthopedic surgeons typically prescribe opioids to manage pain. However, patients undergoing orthopedic procedures have the highest hospital readmission rates for persistent postoperative pain [6].

Previous studies have examined the opioid prescription habits of orthopedic surgeons and have revealed that most surgeons prescribe opioids in excess. A 2018 study by Sabatino et al. reviewed the prescription data for 1,199 procedures [7]. The median numbers of 5 mg oxycodone prescribed were 90 pills for total hip arthroplasty, 90 pills for total knee arthroplasty (TKA), 20 pills for endoscopic carpal tunnel release, 80 pills for arthroscopic rotator cuff repair, and 80 pills for lumbar decompression. Among the opioids prescribed, 61% were unused [7]. In a 2018 systematic review by Feinberg et al., 11 patient surveys reported that patients used only 5.6%-59% of their opioid prescriptions [8]. Moreover, patients who take more opioids after operative fracture repair may report more significant pain and are less satisfied with their pain control [9]. To reduce opioid usage and opioid overprescription, surgeons should incorporate non-pharmacological or alternative modalities into their pain management regimen.

Alternative pain management modalities include acupuncture, electrotherapy, transcutaneous electrical nerve stimulation, and cannabis [10-12]. Although there has been increased interest in non-pharmacological treatments, current research has provided conflicting evidence on virtual reality (VR) therapy [11]. VR has become increasingly popular in research on multimodal pain management [13-15]. VR involves an immersive experience in a 3D environment that people can experience through a head-mounted device with visual input, body-tracking sensors, and headphones for audio input [15]. VR therapy incorporates scenic and interactive scenarios that allow people to believe they are part of a virtual world [16].

VR therapy can be an effective complementary or adjunct modality for postoperative pain management [17]. The thought is that in VR therapy, patients are immersed into a virtual world, taking attention away from the perception of pain. This involves simulations that make use of various combinations of interaction between devices and sensory display systems [17]. This study aims to review relevant current literature on the use and efficacy of VR therapy for postoperative management. We will discuss its effectiveness in pain and anxiety management, opioid reduction, and its implication in orthopedic surgery.

## Review

Method

For this review, a literature search of web-based scientific databases was performed using PubMed. Search criteria included the following: "Virtual reality" AND "postoperative" AND "pain management,” “Virtual reality pain management orthopedic”, "virtual reality" AND "orthopedic" AND "pain,” "virtual reality" AND "opioid" AND "surgery.” Articles published between 2017 and 2023 were selected, including clinical trials, randomized controlled trials (RCTs), meta-analyses, and systematic reviews. Studies were excluded if participants were not involved in any procedures. Studies that used augmented reality and superimposed images in a current environment instead of VR were also excluded. A total of 37 articles were produced from our search. After exclusion criteria and duplicates were removed, this review included 20 studies. 

VR pain control through distraction

According to gate control theory, pain is a negative sensation caused by noxious stimuli [18]. Interneurons in the spinal cord modulate and attenuate the intensity of nociceptive stimuli. How someone perceives pain depends on awareness, emotions associated with pain, and previous memories of pain [18]. According to McCaul et al., humans have a limited capacity for attention, and pain is a sensation that requires attention. Distraction can attenuate nociceptive stimuli and reduce pain perception [19]. In VR therapy, an engaging environment acquires full attention and concentration, which can diminish painful stimuli [15]. 

Pain and anxiety management

Recent studies have tested the effectiveness of VR therapy in reducing pain and anxiety before and after the procedure with conflicting results. Ejilers et al. studied 191 children (ages 4-12 years) who underwent elective maxillofacial, dental, or ENT surgeries [20]. This study utilized VR to expose children to what they can expect on the day of surgery. The virtual environment walks participants through checking in, meeting the anesthesiologist, and arriving at the operating table. Researchers assessed children's preoperative anxiety with the modified Yale Preoperative Anxiety Scale during induction of anesthesia at three different times. Children also self-reported their anxiety level on a visual analog scale (VAS) prior to anesthesia and after surgery. They found no significant differences between the VR and control groups in reducing pain and anxiety during their respective procedures.

However, children undergoing more painful surgical procedures (i.e., ENT surgeries) required less morphine after the VR exposure [20]. A limitation of this study was that the researchers excluded the most anxious children who received anti-anxiety medications. Therefore, this study may include participants who are more tolerant of stressful situations. Children in the VR group requiring less morphine after more painful procedures indicate that VR may be more effective with complex procedures. Incorporating VR in postoperative care may reduce breakthrough pains that require stronger pain medications.

Similarly, Rousseaux et al. found no significant differences in anxiety and pain parameters among 100 adults (>18 years old) undergoing cardiac procedures such as coronary artery bypass graft, mitral valve replacement, and aortic valve replacement [21]. This study divided participants into four groups: hypnosis, VR, hypnosis plus virtual reality (VRH), and control. Participants in the VR group watched the 3D landscape of a cabin with a lake and mountains in the background. Participants in the VR group had decreased anxiety from baseline before surgery. However, their anxiety increased after the surgery and remained elevated. Patients in the hypnosis group had significantly higher anxiety than those in the VRH group. The VRH group had decreased anxiety before and after the surgery. For pain, the VR group reported higher pain levels than the control group after the surgery. This study did not conclude whether one treatment is superior to the other for anxiety and pain management. People dropped out due to disinterest in hypnosis or VR. Participants with a better understanding of technology would benefit more from VR.

Haisley et al. studied patients undergoing laparoscopic foregut surgeries (fundoplication, paraesophageal hernia repair, esophageal myotomy, and pyloroplasty) [22]. This study included 52 patients with a median age of 65. Patients in the VR group used the "Flow VR - Meditation for Modern Life" application, which provided six guided meditation exercises. Patients were asked to rate how often they felt anxiety and pain within the first 24 hours postoperatively. They found that VR mindfulness was not statistically significant in reducing pain and anxiety. This study's small sample size of 52 patients and a high median age of 65 years make generalizability to another population difficult. The use of VR mindfulness is also a passive activity. If the results were significant, it would be difficult to conclude whether people experienced less pain and anxiety due to VR therapy or mindfulness itself [22].

Several studies have reported a significant decrease in pain control after VR therapy. Karaveli et al. in 2021 examined 60 adults (aged >18 years) who underwent colonoscopies without sedation [23]. Participants watched distracting images through an Android mobile phone placed in Cardboard Super Flex Goggles. The VR application, "A Walk on the Beach," was shown during the colonoscopy procedure. They found that those who used VR reported decreased pain during the procedure (p<0.03). There were no statistically significant findings regarding anxiety reduction [23]. Although insignificant, the patient's heart rates were elevated before and after colonoscopy. From this study, VR therapy is more effective at reducing patient's subjective experiences with pain. The elevated heart rate and anxiety signify the physiologic component of pain where distraction was not sufficient to resolve. However, the accessibility of VR applications through mobile devices makes it a worthwhile adjunct therapy during procedures.

Pandrangi et al. investigated postoperative pain management among hospitalized patients after head and neck surgery [24]. They compared pain outcomes of 30 patients (mean age, 58 years) assigned to a VR active game or a similar 2D game. People in the active VR group participated in a 15-minute VR game, "Angry Birds VR: Isle of Pigs." The other group played the same game, a 2D smartphone version. They found that the VR group clinically reduced postoperative pain in patients immediately after the procedure and for up to 4 hours. This study showed that interactive VR games can provide long-term pain reduction [24]. This study's small sample size is a notable limitation. The reduction in pain does show that interactive VR has a place in inpatient pain management after head and neck surgery. At most, patients participating in VR gaming could have better entertainment while recovering in the hospital. 

Spiegel et al. performed a prospective, randomized, comparative effectiveness trial in 30 patients admitted to the hospital for orthopedic, gastrointestinal, or psychiatric reasons [25]. They compared the therapeutic benefits of VR versus in-room "health and wellness" television channels. The VR group significantly reduced pain scores (p<0.04). The effect of VR was larger when analyzing the subgroup of patients with the most severe baseline pain scores (pain rated ≥7/10) (p=0.02). Pain scores at 48- and 72-hour periods before and after the intervention were significantly reduced for the VR group when adjusted for time, study group (VR vs. control), age, sex, and type of pain (somatic vs. visceral) [25].

Huang et al. wrote a systemic review and meta-analysis evaluating whether VR therapy affects subjective pain, anxiety, and pain tolerance [26]. Of 31 RCTs, the VR group had a VAS pain score that was 1.62 scores less than that of the control group (p <0.001). The VR group also had significantly lower VAS scores in participants younger than 18 years and adults. Additionally, the VR group had lower levels of anxiety and unpleasant pain and spent less time thinking about pain. They concluded VR is more effective for acute pain, such as burn dressing changes, needle-related procedures, postoperative dressing changes, and thermal pain. VR was ineffective against chronic pain due to TKA, chronic lower back pain, or cancer-related pain [26]. One explanation could be that VR did not significantly increase pain tolerance.

A 2022 systematic review and meta-analysis of 16 clinical trials by Lan et al. evaluated VR interventions in 535 burn patients [27]. The clinical trials used "Snow World" and other high-level simulation videos to distract patients. The results showed that the average range-of-motion gain of all joints was significantly higher in the VR group than in the control group (p<0.01). Burn patients in the VR group did not have significantly increased hand grip or pinch strength compared to the control. However, they did have significantly fewer unpleasant feelings and less time to think about pain during rehabilitation than the control group (p<0.01). Finally, the anxiety scores of burn patients in rehabilitation were reported. Results showed that burn patients in the VR group had significantly less anxiety than those in the control group (p=0.03) [27].

Pain management in orthopedics

To date, few studies have examined the effectiveness of VR interventions in postoperative orthopedic procedures. Peng et al.'s systematic review and meta-analysis investigated the clinical effectiveness of VR therapy after TKA [28]. VR-based rehabilitation significantly improved pain (VAS score) within one month (p=0.02). However, the improvement in pain at two and three months was not significant. Four studies that assessed the Western Ontario and McMaster Universities Arthritis Index (WOMAC) scores showed significantly better scores in the VR group than in the control group (p<0.01). The VR group also had a significantly higher Hospital for Special Surgery (HSS) score (evaluating pain, stiffness, and physical function) than the control group (p<0.01) [28].

Fuchs et al. examined VR interventions for pain control after TKA in 55 patients [29]. They treated both groups with conventional physiotherapy and continuous passive motion devices. The study group participants received head-mounted displays that allowed them to project a nature or music film. These interventions were performed on day 1 and day 2 postoperatively. Results showed that both the study and control group had decreased levels of anxiety and pain after the intervention. However, there were no differences in the amount of decreased anxiety and pain, or WOMAC, between the two groups [29].

Hoxhallari et al. investigated 55 patients who had undergone wide-awake local anesthesia hand surgery without a tourniquet [30]. The patients in the VR group viewed a video in which the local anesthetic injection was timed to coincide with the video's climax. The VR patients viewed vivid 3D landscapes, cities, rivers, and underwater oceans during the procedure. Anxiety and fun scores were significantly lower for the VR group. However, pain scores were not significantly different. Among patients with pre-existing anxiety, pain scores were significantly lower in the VR group than in the control group [30].

An RCT by Richey et al. examined 129 pediatric patients involved in outpatient orthopedic procedures. They included outpatient procedures such as cast and pin removal [31]. They also randomized VR groups into Active VR (task completion) and Passive VR (traveling through an environment). There were no significant reductions in pain scores between the VR (active and passive) and control groups before, during, or after the procedure. The fear and anxiety scores were significantly lower in the VR group (active and combined) [31].

Reduction in opioid usage

There have only been a few studies investigating the effectiveness of VR in reducing opioid use in patients undergoing surgical procedures.

Rousseaux et al. compared opioid usage among four groups (hypnosis, VR, VR plus hypnosis, and controls) [21]. They measured the number of patients who required "on-demand" opioid intake during the day post-cardiac procedure if their reported pain exceeded 3/10 on the VAS. No significant differences were observed [21].

Haisley et al. reported that the VR group had an upward trend in narcotic usage within the first 24 hours post-op but this number eventually balanced out over hospitalization (p=0.10) [22]. Although not significant (p=0.52), they also found comparable rates of postoperative utilization and refills of narcotics, with the VR group having slightly fewer narcotic refills than the control group (five in the VR group and seven in the control group). The VR group also underwent more complex procedures [22]. In this study, VR meditation may not sufficiently provide the distraction that could mask the pain of more complex procedures. 

Spiegel et al. found no significant differences between VR and control groups [25]. However, a limitation of the study is that they did not instruct nurses to substitute VR for opioids. This misunderstanding could cause nurses to dispense opioids on demand per surgeon's order [25]. McCune et al. performed an RCT on 30 patients undergoing laparoscopic hysterectomy [32]. The people in the VR group selected between meditations with natural landscapes or different scenery with relaxing music or nature sounds. They reported no significant differences in opioid usage between VR and control groups. Patients in the VR group reported lower VAS pain but the results were not significant. This study's small sample size of 30 patients was a significant limitation [32].

Few studies have shown that VR therapy is effective in reducing postoperative opioid use. In Pandrangi et al., opioids and nonopioid medication were documented 8 hours after the VR intervention [24]. They found a clinically meaningful decrease in opioid usage 4 hours and 8 hours before and after the VR intervention [24]. This finding suggests that interactive VR games may be clinically effective in the long-term reduction of opioid use after head and neck surgery. The clinically meaningful reduction in self-reported pain facilitates the reduction in opioid use.

Payne et al. conducted a single-center crossover pilot trial of 35 women (above 18 years old) undergoing gynecologic laparoscopic procedures [33]. They compared the opioid requirements of participants using active and passive VR interventions. Two different VR contents, "Sky Lights 2" and "Cosmic You," were delivered through the Oculus Go headset. The passive intervention content, "Sky Lights 2", had participants lay down in a quiet field and stare at a starry night with Chinese lanterns above. The active intervention content, "Sky Lights 2", allowed participants to participate in a guided meditation. Pain scores were not statistically different (p=0.071). However, the group that received active VR intervention required significantly fewer opioids at 0-10 min: 0 for active VR vs. 3 for passive VR (p=0.04) [33]. 

VR therapy's effectiveness in reducing opioid usage remains inconclusive. More studies showed there was no significant reduction in opioid use. These studies vary in the complexity of procedures. Complex procedures will produce more pain than non-complex procedures. Consequently, they may use opioids more during the first moment they experience breakthrough pain.

Active VR versus passive VR activities

Active VR therapy is more effective than passive VR therapy in improving postoperative recovery [34]. Active VR therapy resembles video games and requires active participation. Passive VR therapy requires listening or observing a landscape, environment, or guided meditation/mindfulness. Studies that included active VR therapy reported a significant decrease in pain [24,25,28,33].

For opioid reduction, the Spiegal et al. study did not see significant differences in opioid reduction between the VR group and the control group [25]. However, patients were randomized to either passive or active VR activity, which could interfere with the results [25]. Active VR interventions have shown a significant reduction in opioid usage [25,34]. Studies using passive VR interventions did not find significant postoperative opioid reduction [22,23,32].

During cast removal, active VR is more effective in decreasing anxiety than standard care [31]. In contrast, other studies reviewed in this study showed that participants who were exposed to passive VR therapy (nature, landscape, or music film) for pain and anxiety after procedures did not show significant differences in anxiety when compared with the control group [21,29]. Similarly, Karaveli et al. used passive VR (portraying images) and found no significant decrease in anxiety or pain post-procedure [23]. In Eijlers et al.'s study, the VR intervention was a child-friendly interactive environment in the operating room. They found no significant differences between the groups in preoperative anxiety, pain, emergence delirium, and parental anxiety in pediatric patients [20]. 

Active VR activities provide greater stimulation that engages the user's mind and body. VR activities force participants to engage with the game rather than be passive observers. Due to the subjective nature of pain, controlling the processing of pain is crucial in pain management and opioid reduction. In contrast to pain, anxiety is a psychological, behavioral, and physiological response to an adverse or unexpected outcome [35]. In the pediatric population, children undergoing procedures may have higher anxiety than adults due to the fear and uncertainty of the hospital setting. Interactive games such as VR are readily available to alleviate anxiety with few adverse outcomes [36].

Previous studies have supported the use of interactive VR interventions over passive VR. Future studies should also investigate the effect of interactive versus passive VR on postoperative patient outcomes.

## Conclusions

This literature review showed that VR can effectively manage pain after minor and major surgical procedures. Given the support provided by VR in reducing anxiety, pain, and opioid usage in various surgical specialties, this novel technology is promising as an adjunct therapy in orthopedics. Several studies included in this study should be interpreted with caution due to its small sample size. Results could also vary based on whether participants enjoy using VR due to the participants' various ages. Older participants may enjoy technology less than younger participants. Furthermore, the VR headset could also be heavy and distracting, which may negatively affect anxiety and pain measurements. More studies are needed to investigate the clinical benefit of using interactive VR in orthopedics and understand its role in postoperative management.
